# Pharmacogenetic profiling via genome sequencing in children with medical complexity

**DOI:** 10.1038/s41390-022-02313-3

**Published:** 2022-09-27

**Authors:** Amy Pan, Sierra Scodellaro, Tayyaba Khan, Inna Ushcatz, Wendy Wu, Meredith Curtis, Eyal Cohen, Ronald D. Cohn, Robin Z. Hayeems, M. Stephen Meyn, Julia Orkin, Jaskiran Otal, Miriam S. Reuter, Susan Walker, Stephen W. Scherer, Christian R. Marshall, Iris Cohn, Gregory Costain

**Affiliations:** 1 Genetics and Genome Biology, Research Institute, The Hospital for Sick Children, Toronto, ON Canada; 2 Program in Translational Medicine, The Hospital for Sick Children, Toronto, ON Canada; 3 Division of Clinical Pharmacology and Toxicology, Department of Paediatrics, The Hospital for Sick Children, University of Toronto, Toronto, ON Canada; 4 Department of Paediatrics, University of Toronto, Toronto, ON Canada; 5 Division of Paediatric Medicine, The Hospital for Sick Children, Toronto, ON Canada; 6 Child Health Evaluative Sciences, Research Institute, The Hospital for Sick Children, Toronto, ON Canada; 7 Institute of Health Policy Management and Evaluation, University of Toronto, Toronto, ON Canada; 8 Edwin S.H. Leong Centre for Healthy Children, University of Toronto, Toronto, ON Canada; 9 Division of Clinical and Metabolic Genetics, The Hospital for Sick Children, Toronto, ON Canada; 10 Department of Molecular Genetics, University of Toronto, Toronto, ON Canada; 11 Centre for Genetic Medicine, The Hospital for Sick Children, Toronto, ON Canada; 12 Center for Human Genomics and Precision Medicine, University of Wisconsin, Madison, WI USA; 13 The Centre for Applied Genomics, The Hospital for Sick Children, Toronto, ON Canada; 14 Genome Diagnostics, Department of Paediatric Laboratory Medicine, The Hospital for Sick Children, Toronto, ON Canada; 15 Laboratory Medicine and Pathobiology, University of Toronto, Toronto, ON Canada

## Abstract

**Background:**

Children with medical complexity (CMC) are a priority pediatric population, with high resource use and associated costs. Genome-wide sequencing is increasingly organized for CMC early in life as a diagnostic test. Polypharmacy becomes common as CMC age. Clinically relevant pharmacogenetic (PGx) information can be extracted from existing genome sequencing (GS) data via GS-PGx profiling. The role of GS-PGx profiling in the CMC population is unclear.

**Methods:**

Prescribed medications were extracted from care plans of 802 eligible CMC enrolled in a structured Complex Care Program over a 10-year period. Drug-gene associations were annotated using curated Clinical Pharmacogenetics Implementation Consortium data. GS-PGx profiling was then performed for a subset of 50 CMC.

**Results:**

Overall, 546 CMC (68%) were prescribed at least one medication with an established PGx association. In the GS-PGx subgroup, 24 (48%) carried variants in pharmacogenes with drug-gene guidelines for one or more of their current medications. All had findings of potential relevance to some medications, including 32 (64%) with variants in *CYP2C19* that could affect their metabolism of proton-pump inhibitors.

**Conclusion:**

GS-PGx profiling at the time of diagnostics-focused genetic testing could be an efficient way to incorporate precision prescribing practices into the lifelong care of CMC.

**Impact:**

- Polypharmacy and genetic test utilization are both common in children with medical complexity.
- The role of repurposing genome sequencing data for pharmacogenetic profiling in children with medical complexity was previously unclear.
- We identified a high rate of medication use with clinically relevant drug-gene associations in this priority pediatric population and demonstrated that relevant pharmacogenetic information can be extracted from their existing genome sequencing data.
- Pharmacogenetic profiling at the time of diagnostics-focused genetic testing could be an efficient way to incorporate precision prescribing practices into the lifelong care of children with medical complexity.

## Introduction

Children with medical complexity (CMC) are a well-studied, clinically defined group in pediatrics.^1–3^ They typically have at least one severe chronic condition, technology dependence, multiple subspecialist involvement, and extensive care coordination needs.^1^ Polypharmacy is common in CMC^4–6^ and was recently identified as a high-priority research area by clinicians and families.^7^ Adverse drug reactions (ADRs) and drug therapeutic failure are both a cause and a consequence of polypharmacy in children.^5,8^ For many medications, dose requirements, efficacy, and risk for ADRs are partially determined by an individual’s genetic profile.^9,10^ Genotype-guided prescribing is an innovative care model in pediatric medicine^11^ that has not been explored in CMC.

Medications prescribed to children often have established, clinically actionable drug-gene interactions that afford opportunities for genotype-guided prescribing.^12^ A major barrier to the wider adoption of pharmacogenetic (PGx) testing in routine clinical practice is that results are rarely already available at the point of prescription. To address this in CMC, it may be possible to utilize the data already generated from the high rate of uptake of diagnostics-focused genetic testing.^13,14^ Exome sequencing and genome sequencing (GS) are increasingly considered first- or second-tier tests for genetically heterogeneous pediatric presentations,^13,15,16^ which includes most CMC. GS data can be repurposed to identify PGx variation and corresponding phenotypes, in a process that we term GS-PGx profiling.^17^ The utility of GS-PGx profiling in the overall CMC population is unknown.

In this study, we characterized the landscape of polypharmacy in a large cohort of CMC, including annotating medications for known drug-gene associations. We then organized GS-PGx profiling for a subgroup with existing GS data.^13^ We hypothesized that a majority of CMC would be prescribed medications with established PGx associations detectable by GS-PGx profiling.

## Methods

### Defining the study population

CMC were considered for this study if they were followed by the Complex Care Program^18^ at The Hospital for Sick Children (Toronto, Canada) at any point between January 1, 2010, and November 1, 2020. Polypharmacy is not a formal criterion for acceptance into this Complex Care Program. Of the 837 potentially eligible CMC, 35 were excluded because the family: (i) declined Complex Care services after referral or were not followed long enough to have a comprehensive care plan,^14^ and/or (ii) requested a closed chart and declined data sharing. For each of the remaining 802 CMC, phenotype, medication, and genetic testing data were extracted from their electronic medical records and stored in a REDCap database. This retrospective chart review with an accompanying patient consent waiver was approved by the Research Ethics Board at The Hospital for Sick Children. A subgroup of CMC and their family members had existing GS data and subsequently underwent GS-PGx profiling (see below). Additional recruitment details and phenotype data for this subgroup were published previously;^13^ one additional proband and his two parents were sequenced after this publication, for a total of *n* = 50 CMC probands and *n* = 89 parents.

### Annotating medications with PGx associations

Current medications were those listed in each child’s most recent comprehensive care plan.^14^ Medications were categorized by target system(s) and pharmacologic indication(s) using pharmacology indexing databases including Micromedex® (micromedexsolutions.com), and then annotated for PGx associations with “pharmacogenes”.^10^ These drug-gene interactions may prompt clinical action to alter medication plans according to Clinical Pharmacogenetics Implementation Consortium (CPIC®) Dosing Guidelines.^19^ We consulted either guidelines specific to pediatric populations, or guidelines applicable to both adult and pediatric populations. We included drug-gene associations with confirmed CPIC® levels of significance A or B, and/or those with an “Actionable PGx” label as denoted by the Food and Drug Administration.^19^ Natural health products, topical agents, as-needed or PRN medications, and select other compounds were a priori excluded from medication counts (Supplementary Table S1) for the following reasons: (i) precedent set by prior PGx studies,^2,11,12,20^ and (ii) suspected high rate of use and inconsistent reporting in comprehensive care plans.

### GS-PGx profiling

We performed GS using our established methods^20,21^ at The Centre for Applied Genomics (Toronto, Canada). Briefly, we completed short-read GS with the HiSeq X Platform (Illumina Inc) using blood-derived DNA from 50 CMC and their family members. Stargazer (version 1.0.8) was used to call genetic polymorphisms with known PGx associations.^22^ Stargazer detects single nucleotide, indel, and structural variants to output PGx diplotypes of 51 possible pharmacogenes. We selected and obtained results for 16 pharmacogenes with clinically significant associations: *CACNA1S, CFTR, CYP2B6, CYP2C19, CYP2C9, CYP2D6, CYP3A5, DPYD, G6PD, NAT2, NUDT15, RYR1, SLCO1B1, TPMT, UGT1A1*, and *VKORC1*.^23^ Quality control measures included using the family data to ensure Mendelian segregation of specific alleles. We called known *CYP2D6* structural variants (e.g., CYP2D6*5) but not novel structural variants, because of the complexity of the region and consequent technical limitations of Stargazer. Variants that do not follow conventional PGx nomenclature were named with an “S” prefix, as per the naming convention within Stargazer.^22^ PGx diplotypes were then analyzed to determine their corresponding phenotypes (where known). Phenotype categories included a metabolizer status of normal, intermediate, poor, rapid, or ultrarapid, as well as a gene function status of normal, increased, or decreased function. We use the term “PGx variant(s)” in this study to refer to all non-normal metabolizer and gene function statuses.

### Statistical methods

Standard descriptive statistics and graphs were generated using R statistical software, version 4.1.0 (R Foundation for Statistical Computing). Statistical significance was defined as a two-tailed *p* value of <0.05.

## Results

### Genetic test utilization and polypharmacy were both common in CMC

In the cohort of 802 CMC, 447 were males (56%), the median year of birth was 2013 (range, 1999–2020), and the diversity in reported ancestry was reflective of the general population in our region (Supplementary Table S2). Over 88% (*n* = 706) had undergone at least one clinical genetic test. This included 314 CMC (39%) who had genome-wide testing (chromosomal microarray analysis, exome sequencing, and/or GS) before 1 year of age.

The median number of current medications per child was 3 (range, 0–13) after relevant exclusions (Supplementary Table S3), and 558 CMC (70%) were prescribed at least two medications. The most common classes of drugs were gastrointestinal (GI) agents (*n* = 493, 61%) and central nervous system (CNS) agents (*n* = 405, 50%) (Supplementary Table S4). The most common medication sub-categories were gastric acid reducers (*n* = 467, 58%), anticonvulsants (*n* = 346, 43%), antiemetics (*n* = 224, 28%), and asthma (*n* = 205, 26%) (Supplementary Table S5).

### CMC were often prescribed medications with PGx associations

**Fig. 1:**
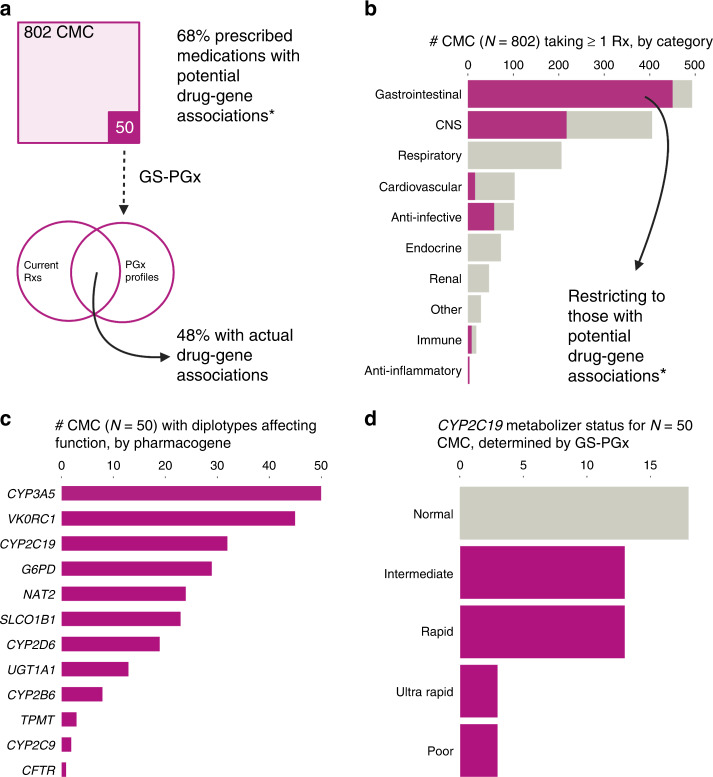
Genome sequencing (GS) for pharmacogenetic (PGx) profiling in children with medical complexity (CMC). **a** Overview of study design and key findings related to currently prescribed medications (Rxs) in a large cohort of CMC. **b** Number of CMC taking medications in each drug category. See main text for details. The cancer drug category (*n* = 3 CMC; Supplementary Table S4) is not shown. **c** Number of CMC with diplotypes affecting metabolism, for each interrogated pharmacogene. See main text for details. The high proportion with PGx variants in *CYP3A5*, *VKORC1*, and *CYP2C19* is consistent with CPIC®-reported frequencies in the general population. **d** Metabolizer status phenotypes for *CYP2C1*.

Overall, 546 (68%) of 802 CMC were currently prescribed at least one medication with an established PGx association (Fig. 1a). This included 450 CMC (56%) for one or more GI agents (e.g., 347 CMC were prescribed omeprazole, which interacts with *CYP2C19*) and 217 (27%) for one or more CNS agents (e.g., 117 CMC were prescribed clobazam, which interacts with *CYP2C19*) (Fig. 1b). The proportions of CMC prescribed medications with PGx associations, by drug category and sub-category, are listed in Supplementary Tables S4 and S5, respectively.

Results were similar in the subgroup that underwent GS-PGx profiling (Fig. 1a), with 39 of 50 (78%) currently prescribed at least one medication with an established PGx association. GI, CNS, and respiratory agents with PGx associations were all in use by ten or more of these CMC (Supplementary Table S4). The two medication sub-categories with the highest PGx relevance were gastric acid reducers (specifically, the proton-pump inhibitors (PPIs) omeprazole, lansoprazole, and pantoprazole; currently prescribed to a total of 31 CMC) and anticonvulsants (specifically, carbamazepine, clobazam, lamotrigine, oxcarbazepine, and valproic acid; currently prescribed to a total of 16 CMC). Half (8 of 16 CMC) were prescribed two or more of these anticonvulsants.

### GS-PGx profiling identified findings in CMC relevant to their current medications

The median number of PGx variants per CMC was 5 (range, 2–8). GS-PGx findings by pharmacogene are summarized in Fig. 1c. For example, 32 (64%) of the 50 CMC had *CYP2C19* diplotypes that could impact dosing for some of the most prescribed medications in CMC (i.e., PPIs): 13 were intermediate metabolizers, 13 were rapid metabolizers, 3 were ultrarapid metabolizers, and 3 were poor metabolizers (Fig. 1d). The burden of PGx variants amongst the parents of CMC was similar, with a median of 5 (range, 1–8) per parent (Supplementary Table S6).

**Fig. 2:**
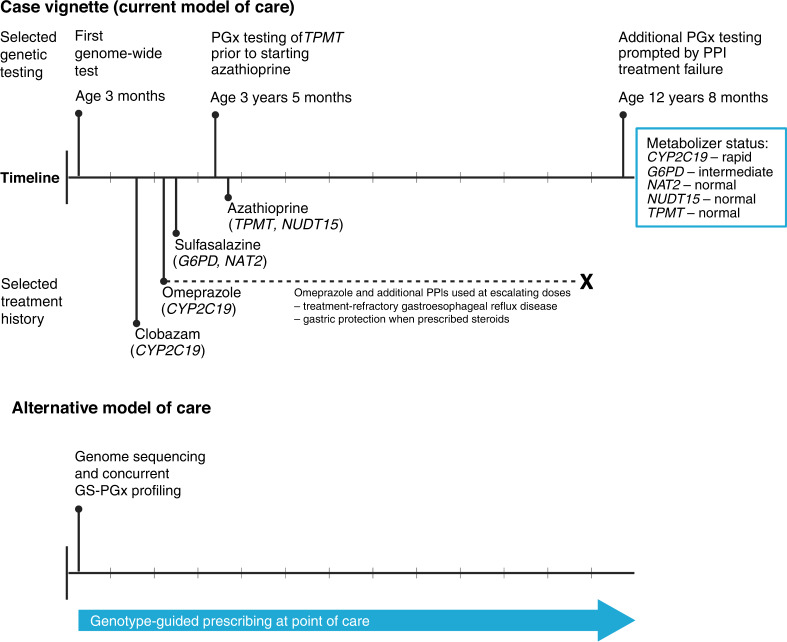
Case vignette illustrating the current model of care and potential role for pre-emptive pharmacogenetic (PGx) profiling via genome sequencing (GS) in a child with medical complexity. This child underwent their first genome-wide test at age 3 months because of neurological signs and symptoms, and was ultimately diagnosed with a rare X-linked genetic syndrome. Clobazam was started as an anti-seizure medication after the age of 1 year. Proton-pump inhibitors (PPIs) were prescribed for two different indications: gastroesophageal reflux disease, and gastric protection in the context of treatment with corticosteroids. The latter, along with sulfasalazine and azathioprine, was used to treat inflammatory bowel disease.

Cross-referencing GS-PGx profiling results with current medication lists identified 48% of CMC (24 of 50) with at least one applicable drug-gene association (Fig. 1a and Supplementary Table S7). This included 5 CMC (10%) who were prescribed two or more different medications with each impacted by that child’s variation in a different pharmacogene. A major contributor to these findings was the association between *CYP2C19* diplotypes and metabolism of PPIs (Fig. 1d and Supplementary Table S7). There were 18 CMC with metabolizer statuses currently affecting a prescribed medication: 9 intermediate, 7 rapid, 1 ultrarapid, and 1 poor. Eight additional CMC were not currently prescribed a PPI but had *CYP2C19* diplotypes indicating a rapid or ultrarapid metabolizer status. Review of lifetime medication histories revealed that at least five of eight had trialed a PPI in the past, suggesting a missed opportunity for genotype-guided prescribing. Figure 2 depicts a representative case vignette.

## Discussion

These results indicate that CMC are often prescribed medications with established PGx associations and dosing guidelines. PGx diplotypes can be reliably extracted from GS data. Genetic test utilization is already high in CMC, and exome sequencing and chromosomal microarray analysis are expected to be replaced by GS in the coming years.^13,15,16,24^ Genotype-guided prescribing can have the greatest impact when initiated at a child’s first point of contact with the healthcare system,^25^ with the caveat that some findings may not be applicable until after the neonatal period or infancy.^25,26^ GS-PGx profiling at the time of initial etiologic-based testing therefore warrants strong consideration in CMC (Fig. 2).

### CMC are a priority population for trialing genotype-guided prescribing in pediatrics

Unique characteristics of CMC provide the rationale for positioning them at the leading-edge of broad PGx testing amongst children and adolescents. Neurological impairment, multi-organ system disease, and multiple subspecialist prescribers are all common, and these factors can complicate clinical assessment of treatment response/failure and side effects. Medication use patterns are shared across CMC because of the development of similar comorbidities over time, particularly in those with severe neurological impairment.^27^ Medication dosing that is unsuited to the individual’s genetic profile may place additional stress on patients and their families.^5,28^ PGx data can also provide insight into drug-drug interactions, a common concern in polypharmacy.^29^ The prevalence of polypharmacy in this study cohort was comparable to adults with psychiatric illness and the elderly, populations where PGx profiling is most common and best established.^30^ As expected, PGx variants were as common in CMC as they are in the general population.^19,31^ These observations suggest a strong potential for GS-PGx profiling to alter medication choices and dosages for CMC, particularly with PPI selection and dosing for rapid and ultrarapid metabolizers in accordance with published CPIC® guidelines.^32^

We propose to integrate GS-PGx as a secondary use of GS data being generated for diagnostic purposes. Efforts to clinically validate this approach are in progress at our center and others.^33^ Automated reporting will facilitate its application.^34^ With GS-PGx being a low-cost adjunct analysis to an already planned GS experiment, there is the potential for cost-effectiveness. Important barriers and knowledge gaps remain, however. There is a relative paucity of data in the pediatric age range.^26^ Certain “established” PGx associations cannot be extrapolated to neonates because of key physiological differences (e.g., immature enzyme expression).^25^ Clinical implementation of GS-PGx will need to be accompanied by continuing professional education and other initiatives to ensure appropriate interpretation of findings at the bedside.^9^

### Advantages and limitations

We used a cross-sectional design that captured current medication use at a single point of time. As illustrated by our post hoc review of lifetime medical records for those with *CYP2C19* rapid and ultrarapid metabolizer statuses, we have likely underestimated both the scope of polypharmacy and the potential role of PGx. Our a priori exclusion criteria with respect to medication counts were also conservative; many as-needed or PRN medications have well-established drug-gene associations (e.g., ibuprofen and *CYP2C9*). We were unable to determine conclusively whether current or past medication use was influenced by ADRs.

We were conservative in considering only drug-gene associations at CPIC® levels of significance A and B only. Many drugs remain under review for clinical significance and have not yet been assigned a CPIC® significance level (resulting in “Provisional” status). Provisional drug-gene pairs like valproic acid and *POLG*, or fluticasone propionate and *CRHR1*, could become particularly relevant to CMC given the high rate of associated medication use. Compared with targeted genotyping approaches, GS-PGx profiling was able to identify uncommon PGx alleles in this ethnically diverse cohort (e.g., CYP2C9*3, CYP2D6*20; Supplementary Table S8). However, interpretation of ultra-rare and novel genetic variants in pharmacogenes, which can be detected by GS, remains challenging.^35^ *HLA* genotyping remains beyond the analytical scope of GS-PGx for now because of the complexity of that genomic region.^17^

Last, we acknowledge the ongoing technical limitations of Stargazer. There continue to be challenges in predicting rare alleles and resolving star alleles in instances of heavy sequence noise and complex structural variation.^36^ For example, UGT1A1*28 is a short tandem repeat in a non-coding region that cannot be reliably detected because of the complexity of regional structural variation. At present, Stargazer is the bioinformatics tool that is most readily available and widely used to perform GS-PGx profiling.^33^ Improvements in both GS and PGx profiling are expected over time.

## Conclusion

GS-PGx profiling at the time of diagnostics-focused genetic testing could be an efficient way to incorporate precision prescribing practices into the lifelong care of CMC. These data provide the impetus for further study of GS-PGx, to determine therapeutic, patient outcome, and societal efficacies in clinical practice.^37^

## Supplementary information

Supplementary File

Table S3

## Data Availability

Most of the data generated or analyzed during this study are included in this published article and its supplementary information files. The remaining data are available from the corresponding author upon reasonable request.
