# Supplementary material for: Pharmacogenetic profiling via genome sequencing in children with medical complexity
Source: Pediatr Res. 2022 Sep 27;93(4):905–10. doi: 10.1038/s41390-022-02313-3 (PMC10033400; doi:10.1038/s41390-022-02313-3)
Supplement: Supplementary file 1 — Supplementary File [file 41390_2022_2313_MOESM1_ESM.docx]

**SUPPLEMENTAL FILE**

**Pharmacogenetic profiling via genome sequencing in children with medical complexity**

Amy Pan, Sierra Scodellaro, Tayyaba Khan, Inna Ushcatz, Wendy Wu, Meredith Curtis, Eyal Cohen, Ronald D. Cohn, Robin Z. Hayeems, M. Stephen Meyn, Julia Orkin, MD, Jaskiran Otal, Miriam S. Reuter, Susan Walker, Stephen W. Scherer, Christian R. Marshall, Iris Cohn, Gregory Costain

**Table of contents:**

Table S1………………………………………………………………………………………2

Table S2………………………………………………………………………………………4

[Table S3 – see separate .xls file]

Table S4………………………………………………………………………………………5

Table S5………………………………………………………………………………………6

Table S6………………………………………………………………………………………7

Table S7………………………………………………………………………………………8

Table S8………………………………………………………………………………………9

**Table S1. Medications and supplements excluded *a priori* from medication counts.**

| Acetylsalicylic acid |
| --- |
| Adapalene-benzoyl peroxide |
| Apraclonidine |
| Atropine |
| Benzoyl peroxide-clindamycin |
| Bimatoprost |
| Brinzolamide-brimonidine |
| Brinzolamide-timolol |
| Calcium supplements (various formulations) |
| Cannabis-derived compounds (various formulations) |
| Carboxymethylcellulose sodium |
| Chicory root |
| Chloral hydrate |
| Chromium supplements (various formulations) |
| Copper supplements (various formulations) |
| Cranberry |
| Creatine monohydrate |
| Cyclopentolate |
| D-amino acids |
| Desonide |
| Diphenhydramine |
| Dorzolamide |
| Dorzolamide-timolol |
| Echinacea |
| Eucerin® |
| Fluorometholone |
| Fusidic acid (various formulations) |
| Ginseng root extract |
| Glycerin |
| Gramicidin-polymyxin B |
| Hypromellose |
| Ibuprofen |
| Intravenous immune globulin |
| Iodine supplement |
| Iron supplements (various formulations) |
| Latanoprost |
| Latanoprost-timolol |
| Levocarnitine |
| Lubricant eye drops (various formulations) |
| Magnesium supplements (various formulations) |
| Manganese supplement |
| MCT oil |
| Melatonin |
| Methyl cellulose |
| Midazolam |
| Mineral oil |
| Omega-3 supplements (various formulations) |
| Polyethylene glycol 3350 |
| Peptan® collagen peptides |
| Potassium supplement (various formulations) |
| Probiotics (various formulations) |
| Riboflavin supplement |
| Salbutamol |
| Salicylic acid |
| Selenium supplement |
| Silver sulfadiazine |
| Sodium supplements (various formulations) |
| Taurine supplement |
| Terbinafine |
| Thiamine supplement |
| Topical steroids (various formulations) |
| Tube feeding formulas and additives (various formulations) |
| Travoprost |
| Turmeric root extract |
| Ubidecarenone |
| Vitamins & vitamin analogues not listed above (various formulations) |
| White petroleum-mineral oil |
| Zinc oxide |
| Zinc supplements (various formulations) |

**Table S2. Demographic characteristics of the CMC study cohort (N=802).**

|  | GS-PGx subgroup  (n=50) | Remainder  (n=752) | p-value |
| --- | --- | --- | --- |
| **Age** |  |  |  |
| Mean | 9.32 | 8.21 |  |
|  |  |  |  |
| **Year of birth** |  |  | <0.001^a^ |
| Median | 2011 | 2013 |  |
| Range | 1999-2018 | 2002-2020 |  |
|  | | | |
| **Sex** |  |  | ns^b^ |
| Male | 30 (60%) | 417 (55%) |  |
| Female | 20 (40%) | 335 (45%) |  |
|  | | | |
| **Ethnicity**^c^ |  |  | ns^b,d^ |
| South Asian | 9 (18%) | 108 (14%) |  |
| European/White | 8 (16%) | 109 (14%) |  |
| Ashkenazi Jewish | 3 (6%) | 10 (1%) |  |
| Middle Eastern | 3 (6%) | 57 (8%) |  |
| East Asian | 2 (4%) | 54 (7%) |  |
| Latin American | 1 (2%) | 9 (1%) |  |
| Black/African | 0 (0%) | 58 (7%) |  |
| Other/Mixed | 18 (36%) | 186 (25%) |  |
| Unknown | 6 (12%) | 161 (21%) |  |

CMC, children with medical complexity; GS, genome sequencing; ns, non-significant; PGx, pharmacogenetics

^a^Mann-Whitney U test. The GS-PGx subgroup was recruited by November 30, 2018, whilst CMC were included in the overall cohort if they were followed in the Complex Care Program before November 1, 2020 (see main text).

^b^Fisher’s exact test for comparison of proportions.

^c^Self-reported ethnicity documented in the electronic medical record.

^d^The “Unknown” category was excluded from this calculation.

**Table S4. Current prescriptions by main medication category.**

|  | **Total CMC cohort (N=802)** | | | | **GS-PGx subgroup (n=50)** | | | |
| --- | --- | --- | --- | --- | --- | --- | --- | --- |
|  | Any Rx | | Potential drug-gene association | | Any Rx | | Potential drug-gene association | |
| **Rx category**^a^ | n | % | n | % | n | % | n | % |
| Gastrointestinal | 493 | 61 | 450 | 56 | 34 | 68 | 31 | 62 |
| CNS | 405 | 50 | 217 | 27 | 29 | 58 | 19 | 38 |
| Respiratory | 206 | 26 | 0 | 0 | 22 | 44 | 0 | 0 |
| Cardiovascular | 103 | 13 | 16 | 2 | 11 | 22 | 0 | 0 |
| Anti-infective | 101 | 13 | 58 | 7 | 9 | 18 | 4 | 8 |
| Endocrine | 73 | 9 | 0 | 0 | 7 | 14 | 0 | 0 |
| Renal | 47 | 6 | 0 | 0 | 1 | 2 | 0 | 0 |
| Miscellaneous | 29 | 4 | 0 | 0 | 2 | 4 | 0 | 0 |
| Immune | 19 | 2 | 8 | <1 | 1 | 2 | 1 | 2 |
| Anti-inflammatory | 4 | <1 | 4 | <1 | 1 | 2 | 1 | 2 |
| Cancer | 3 | <1 | 1 | <1 | 0 | 0 | 0 | 0 |

CMC, children with medical complexity; CNS, central nervous system; GS, genome sequencing; PGx, pharmacogenetics; Rx, current prescribed medication

^a^Based on prior publications and pharmacology indexing databases including Micromedex® (micromedexsolutions.com).

**Table S5. Current prescriptions by medication subcategory.**

|  | | **Total CMC cohort (N=802)** | | | | **GS-PGx subgroup (n=50)** | | | |
| --- | --- | --- | --- | --- | --- | --- | --- | --- | --- |
|  |  | Any Rx | | Potential drug-gene association | | Any Rx | | Potential drug-gene association | |
| **Rx category**^a^ | **Rx subcategory^a^** | n | % | n | % | n | % | n | % |
| CNS | Anticonvulsant | 346 | 43 | 184 | 23 | 23 | 46 | 16 | 32 |
|  | Antidepressant | 17 | 2 | 9 | 1 | 3 | 6 | 2 | 4 |
|  | Antianxiety | 9 | 1 | 9 | 1 | 0 | 0 | 0 | 0 |
|  | Antipsychotic | 14 | 2 | 12 | 1 | 1 | 2 | 1 | 2 |
|  | Stimulant | 11 | 1 | 1 | <1 | 1 | 2 | 0 | 0 |
|  | Opioid/ analgesic | 34 | 4 | 0 | 0 | 2 | 4 | 0 | 0 |
|  | Neuromuscular | 93 | 12 | 7 | 1 | 6 | 12 | 0 | 0 |
|  | Adrenergic | 51 | 6 | 16 | 2 | 8 | 16 | 1 | 2 |
|  | Anticholinergic | 14 | 2 | 0 | 0 | 1 | 2 | 0 | 0 |
|  | Other | 2 | <1 | 0 | 0 | 0 | 0 | 0 | 0 |
| Cardiovascular | Anti-hypertensive | 70 | 9 | 10 | 1 | 4 | 8 | 0 | 0 |
|  | Anti-hypotensive | 1 | <1 | 0 | 0 | 0 | 0 | 0 | 0 |
|  | Anti-coagulant | 23 | 3 | 3 | <1 | 5 | 10 | 0 | 0 |
|  | Statins | 1 | <1 | 1 | <1 | 0 | 0 | 0 | 0 |
|  | Anti-arrhythmic | 5 | <1 | 5 | <1 | 0 | 0 | 0 | 0 |
|  | Other | 18 | 2 | 1 | <1 | 3 | 6 | 0 | 0 |
| Gastrointestinal | Gastric acid reducer/PPI | 467 | 58 | 449 | 56 | 31 | 62 | 31 | 62 |
|  | Anti-diarrhea | 5 | <1 | 0 | 0 | 1 | 2 | 0 | 0 |
|  | Anti-constipation | 61 | 8 | 0 | 0 | 7 | 14 | 0 | 0 |
|  | Anti-emetic | 224 | 28 | 6 | 1 | 13 | 26 | 0 | 0 |
|  | Other | 15 | 2 | 0 | 0 | 2 | 4 | 0 | 0 |
| Respiratory | Anti-asthma | 205 | 26 | 0 | 0 | 20 | 40 | 0 | 0 |
|  | Other | 4 | <1 | 0 | 0 | 2 | 4 | 0 | 0 |
| Renal | Diuretic | 36 | 4 | 0 | 0 | 0 | 0 | 0 | 0 |
|  | Other | 11 | 1 | 0 | 0 | 1 | 2 | 0 | 0 |
| Endocrine | Hormonal/ thyroid | 50 | 6 | 0 | 0 | 5 | 10 | 0 | 0 |
|  | Corticosteroid | 15 | 2 | 0 | 0 | 2 | 4 | 0 | 0 |
|  | Diabetic | 8 | 1 | 0 | 0 | 0 | 0 | 0 | 0 |
| Cancer | Anti-cancer | 3 | <1 | 0 | 0 | 0 | 0 | 0 | 0 |
| Anti-infective | Anti-biotic | 87 | 11 | 57 | 7 | 9 | 18 | 4 | 8 |
|  | Anti-fungal | 14 | 2 | 1 | 0 | 0 | 0 | 0 | 0 |
|  | Anti-viral | 5 | <1 | 0 | 0 | 0 | 0 | 0 | 0 |
| Anti-inflammatory | Anti-inflammatory | 4 | <1 | 4 | 0 | 1 | 2 | 1 | 2 |
| Immune | Immunosuppressant | 13 | 2 | 8 | 1 | 1 | 2 | 1 | 2 |
|  | Other | 7 | <1 | 0 | 0 | 0 | 0 | 0 | 0 |
| Miscellaneous | Anti-histamine | 16 | 2 | 0 | 0 | 1 | 2 | 0 | 0 |
|  | Genetic/metabolic | 14 | 2 | 0 | 0 | 1 | 2 | 0 | 0 |

CMC, children with medical complexity; CNS, central nervous system; GS, genome sequencing; PGx, pharmacogenetics; PPI, proton pump inhibitor; Rx, current prescribed medication

^a^Based on prior publications and pharmacology indexing databases including Micromedex® (micromedexsolutions.com).

**Table S6. GS-PGx profiling results for n=89 parents of CMC, by interrogated pharmacogene.**

| **Pharmacogene** | **PGx variants** | |
| --- | --- | --- |
|  | **n** | **%** |
| *CACNA1S* | 0 | 0 |
| *CFTR* | 0 | 0 |
| *CYP2B6* | 8 | 9 |
| *CYP2C19* | 43 | 48 |
| *CYP2C9* | 1 | 1 |
| *CYP2D6* | 22 | 25 |
| *CYP3A5* | 85 | 96 |
| *DPYD* | 0 | 0 |
| *G6PD* | 43 | 48 |
| *NAT2* | 49 | 55 |
| *NUDT15* | 0 | 0 |
| *RYR1* | 0 | 0 |
| *SLCO1B1* | 43 | 48 |
| *TPMT* | 10 | 11 |
| *UGT1A1* | 23 | 26 |
| *VKORC1* | 79 | 89 |

CMC, children with medical complexity; GS, genome sequencing; PGx, pharmacogenetics

**Table S7. Proportions of n=50 CMC found by GS-PGx profiling to have actual drug-gene association, by current prescribed medication.**

| **Medication** | **Drug subcategory** | **Associated gene(s)** | **Actual drug-gene associations** |
| --- | --- | --- | --- |
| Omeprazole | PPI | *CYP2C19* | 14 (28%) |
| Clobazam | Anticonvulsant | *CYP2C19* | 6 (12%) |
| Lansoprazole | PPI | *CYP2C19* | 2 (4%) |
| Pantoprazole | PPI | *CYP2C19* | 2 (4%) |
| Trimethoprim | Antibiotic | *G6PD*, *NAT2* | 1 (2%) |
| Amitriptyline | Antidepressant | *CYP2C19*, *CYP2D6* | 1 (2%) |
| Sulfasalazine | Anti-inflammatory | *G6PD*, *NAT2* | 1 (2%) |
| Tacrolimus | Immunomodulant | *CYP3A5* | 1 (2%) |
| **All** | - | - | **28 (48%) unique individuals** |

CMC, children with medical complexity; CNS, central nervous system; GS, genome sequencing; PGx, pharmacogenetics; PPI, proton pump inhibitor; Rx, current prescribed medication

| **Pharmacogene** | **Allele(s)**^a,b^ |
| --- | --- |
| *CACNA1S* | *1 |
| *CFTR* | *1, *S37 |
| *CYP2B6* | *1, *2, *4, *5, *6, *22 |
| *CYP2C19* | *1, *2, *17 |
| *CYP2C9* | *1, *2, *3 |
| *CYP2D6* | *1, *2, *4, *5, *9, *20, *33, *35, *39, *41, *68 |
| *CYP3A5* | *1, *3 |
| *DPYD* | *1, *S3, *4, *S4, *5, *S5, *6, *9A, *S9, *S12, *S56 |
| *G6PD* | *1, *10 |
| *NAT2* | *1, *5, *6, *7, *12, *19 |
| *NUDT15* | *1, *3 |
| *RYR1* | *1 |
| *SLCO1B1* | *1, *1B, *5, *14, *15, *17, *35 |
| *TPMT* | *1, *3A, *3C |
| *UGT1A1* | *1, *27, *60 |
| *VKORC1* | *1, *2, *3, *4 |

**Table S8. Alleles identified by GS-PGx profiling with Stargazer in a cohort of n=50 CMC.**

^a^Variants that do not follow conventional pharmacogenetic nomenclature were named with a “S” prefix, as per the naming convention within Stargazer (see text for additional details).

^b^Every haplotype called by Stargazer was manually reviewed and assigned a “activity score” using the CPIC method. The summed diplotype score was then to determine the phenotype classification used in our primary data analyses.
